# A Model-Based Prioritisation Exercise for the European Water Framework Directive

**DOI:** 10.3390/ijerph8020435

**Published:** 2011-02-01

**Authors:** Klaus Daginnus, Stefania Gottardo, Ana Payá-Pérez, Paul Whitehouse, Helen Wilkinson, José-Manuel Zaldívar

**Affiliations:** 1 Institute for Health and Consumer Protection, Joint Research Centre, European Commission, Via E. Fermi 2749, 21027 Ispra, VA, Italy; E-Mails: Klaus.Daginnus@bsu.hamburg.de (K.D.); stefania.gottardo@unive.it (S.G.); Ana.paya-perez@ec.europa.eu (A.P.); 2 Environment Agency, Red Kite House, Howbery Park, Benson Lane, Crowmarsh Gifford, Wallingford, Oxfordshire, OX10 8BD, UK; E-Mails: paul.whitehouse@environment-agency.gov.uk (P.W.); helen.wilkinson@environment-agency.gov.uk (H.W.)

**Keywords:** Water Framework Directive, priority substances, risk assessment

## Abstract

A model-based prioritisation exercise has been carried out for the Water Framework Directive (WFD) implementation. The approach considers two aspects: the hazard of a certain chemical and its exposure levels, and focuses on aquatic ecosystems, but also takes into account hazards due to secondary poisoning, bioaccumulation through the food chain and potential human health effects. A list provided by EU Member States, Stakeholders and Non-Governmental Organizations comprising 2,034 substances was evaluated according to hazard and exposure criteria. Then 78 substances classified as “of high concern” where analysed and ranked in terms of risk ratio (Predicted Environmental Concentration/Predicted No-Effect Concentration). This exercise has been complemented by a monitoring-based prioritization exercise using data provided by Member States. The proposed approach constitutes the first step in setting the basis for an open modular screening tool that could be used for the next prioritization exercises foreseen by the WFD.

## 1. Introduction

The Water Framework Directive (WFD) [1] sets out a strategy to protect against pollution of water; within this strategy, Article 16 of the WFD requires the periodical setting out of a list of priority substances (PS) and priority hazardous substances (PHS)—regarding reduction or phase out—presenting a significant risk to or via the aquatic environment. Substances should be prioritised taking into account: (i) risk assessments carried out under existing chemically-relevant EU (European Union) Directives and Regulations [2–5]; (ii) targeted risk-based assessments focusing on aquatic ecotoxicity and human toxicity via the aquatic environment; (iii) simplified risk-based assessments based on intrinsic hazards, widespread environmental contamination, production volumes and use patterns. For the prioritised substances, Environmental Quality Standards (EQS) referring to the protection of water, sediment or biota need to be developed. By definition [1], an EQS is “*the concentration of a particular pollutant or group of pollutants in water, sediment, or biota that should not be exceeded in order to protect human health and the environment.*”

According to the new Environmental Quality Standards Directive [6], the next revision of the PS list and their EQS must be completed by spring 2011. With this deadline in mind and after extensive discussions and consultations with experts from the EU Member States (MS), the European Commission (EC) decided to run in parallel two complementary priority-setting exercises, one monitoring-based and the other modelling-based. Whereas the monitoring-based approach could rely on experimental data from EU water bodies to provide a picture of the environmental conditions of aquatic ecosystems, the modelling-based approach could be used to detect and assess substances, which are not routinely monitored. This is necessary due to the fact that the majority of wide commercial chemicals with production volumes above 1 t/y are not measured in environmental compartments [7].

**Figure 1.**
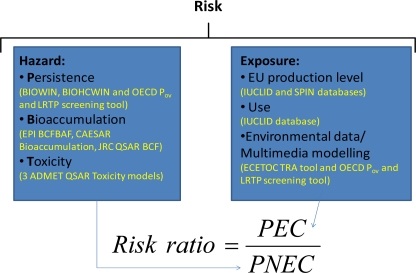
Summary of the prioritisation process based on hazard and environmental exposure and the different tools used when no experimental data was available.

A prioritisation process, Figure 1, should consider two aspects, the first concerns the hazard of a given chemical and the second its exposure levels. In the case of the WFD, the hazard is focused on the aquatic ecosystem, but since the definition of EQS comprises also the protection of human health, the process has also considered hazards due to secondary poisoning, bioaccumulation through the food chain and potential human health effects, e.g. due to the consumption of fish or drinking water. The exposure of a chemical is related to its use and its tonnage, as well as, to its partitioning into environmental media.

The monitoring-based exercise was carried out by INERIS [8–9] using environmental data provided by MS authorities. They compiled and developed a database that has evolved from ∼700,000 data points of 314 substances from 15 countries, to the current monitoring status with ∼1,4000,000 data points of 1,153 substances from 28 countries (EU Member States plus Norway). Then they designed a set of procedures for data processing, treatment; and selection of relevant parameters to consider. Finally, they developed the algorithms for substance’s prioritisation. Based on this methodology, a list of 316 substances for which there were monitoring data from more than three countries in water, sediment, and/or biota was selected as candidates for prioritisation. The Predicted Environmental Concentration (*PEC*) and Predicted No-Effect Concentration (*PNEC*) were calculated and based on the risk ratio, *i.e.*, *PEC/PNEC*, the substances were ranked, and a list of 41 substances was produced with another of 21 substances considering water for human consumption [9].

In this paper, we discuss the modelling-based approach and the steps taken to complete this prioritisation exercise, starting from the selection of chemicals and ending in the application of a risk ratio based on the estimation of *PEC* and *PNEC* values for the substances classified as of highest concern. Even though, the present approach did not consider metals and, in some cases organometallic substances, it provides a comprehensive analysis of chemical prioritisation for the WFD.

## 2. Materials and Methods

The risk scoring discussed in this paper, was adapted from the UK methodology [10], which is based on the integration of hazard and exposure assessments according to specific rules and ranges from 1 to 5. A value of 1 indicates the highest priority (*i.e.*, highest risk) and a value of 5 the lowest risk. The hazard assessment is based on the PBT (Persistence, Bioaccumulation and Toxicity) approach developed in the REACH Guidance [11], whereas the exposure assessment is based on production and use data obtained from the IUCLID and SPIN databases [12]. To rank all substances classified with a score of 1 (78 substances) a PNEC value was estimated using experimental data and QSAR (Quantitative Structure-Activity Relationships) models and a *PEC* value was estimated using both the ECETOC TRA (Targeted Risk Assessment) tool [13] and the Long Range Transport Potential (LRTP) OECD tool [14]. The *PEC/PNEC* risk ratio was then calculated and a ranked list of the 78 substances produced.

### 2.1. Identification of Candidates for Prioritisation

The Starting List of Chemicals (SLoC) was based on inputs from EU Member States (MS), the European Parliament (EP), stakeholders, research consortiums, international organizations and several EU lists of substances of possible concern such as PBT, potential endocrine disruptors, and plant protection products. Specifically the following lists were merged by CAS number:

- All substances in the list of monitoring data provided by MS (922 substances);
- Substances indicated by EU Member States (Denmark, Slovakia, Sweden and United Kingdom) after a general call for substances to be analyzed for prioritisation (712 substances);
- List of substances included by the European Parliament for further investigation (34 substances);
- Lists of substances provided by stakeholders: EEB (European Environmental Bureau) (25 substances), Greenpeace which indicated OSPAR lists of substances for priority action [15] and of substances of possible concern (331 substances), IARW (International Working Group Rhine Waterworks) (25 substances), ESR (Existing Substances Regulation) (141 substances);
- Substances indicated by research consortiums: the NORMAN Association [16] provided a list of Emerging Substances (ES) of concern derived from scientific literature and expert judgment as well as a monitoring database (422 substances);
- Substances indicated by international organizations: OSPAR lists of substances for priority action and of substances of possible concern (331 substances) and ICPDR [17] substances monitored during the second Joint Danube Survey (JDS2) in surface water and sediments (310 substances);
- EU lists of substances from the JRC Website: PBT (TC-NES working group), RAR, IUCLID, ClassLab [18], and potential endocrine disruptor (ED) database [19].

After merging, the initial SLoC list contained 2,034 substances (see *Supplementary information, Excel files*).

### 2.2. Outline of the Modelling-Based Prioritisation Approach

As introduced above, the risk scoring in the modelling-based prioritisation exercise is based on the integration of two separated scores provided after hazard and exposure assessment, plus an additional ranking step based on the *PEC/PNEC* ratios.

The scoring scheme for hazard assessment is calculated as:

(1) $$\text{Total}\;\text{Score}={\text{Score}}_{P}+{\text{Score}}_{B}+{\text{Score}}_{T}+{\text{Score}}_{\text{ED}}$$

where *P* stands for Persistent (no persistence = 0, persistent = 1), *B* for Bioaccumulative (no bioaccumulation = 0, bioacumulative = 1), *T* for Toxic (no toxicity = 0, toxic = 1) and *ED* for being in the Endocrine Disruptors list Categories 1 and 2 (no ED activity = 0, ED = 1). An additional +1 was added to the total score if the substance fulfilled all the screening criteria or if the substance was classified as vPvB (v = very). Therefore, the maximum hazard score is 4 which corresponds to a substance classified as PBT or vPvB, while the minimum score is 0.

Whenever possible, the hazard assessment has been based on experimental data. However, for many substances the available data was scarce or inexistent for a definitive conclusion on PBT or vPvB properties be issued. In those cases, screening methods were used as surrogate information to decide whether a substance may fulfill the PBT or vPvB criteria. These screening methods often include the application of non-testing methods like QSAR—for a detailed discussion on the required conditions on the applicability of QSAR the reader is referred to [20] and references therein. The majority of QSARs have been developed for organic substances; therefore metals and organometallic substances are generally out of the applicability domain of the QSARs employed here and therefore could not be assessed using this approach. Only when experimental data was available the substance was considered. The scoring scheme for exposure assessment is shown in Table 1.

The exposure assessment score is obtained by calculating the annual use as:

(2) Use Assessment=Total Production×Use Index

Each contribution to Equation (2) is explained in Table 2. To avoid a bias in the prioritisation by using the same datasets than in the monitoring-based exercise, it was decided not to include the data provided by Member States to estimate the *PEC* value, but base the assessment in production volumes.

The final Risk scoring is obtained by combining the hazard and exposure assessment results using Table 3 [10].

Finally, all the substances classified with a value of “1” were ranked according to the *PNEC/PEC* ratio. The *PNEC* was obtained from existing experimental values or estimated using QSAR algorithms developed at the JRC (see below), whereas the *PEC* was calculated by applying the ECETOC TRA tool and/or the OECD LRTP multimedia tool to calculate the distribution in water and the following Equation (3):

(3) $$\text{PEC}=\frac{\text{Total}\;\text{Production}\times \text{Use}\;\text{Index}\times \text{Distribution}\;\text{in}\;\text{water}}{25\times 10^{9}}$$

where the value 25 × 10^9^ refers to the water dilution factor in m^3^ year^−1^ proposed in the REACH Guidance [21] and it is applied in the ECETOC TRA tool [13] to estimate the *PEC* value for several wide dispersive outdoor releases scenarios. In principle, this value should provide an upper bound to the *PEC* value.

### 2.3. Hazard Assessment

The hazard assessment was developed as a PBT assessment following the REACH Guidance [11] or according to scientific progress when it was not clear how to distinguish between some categories, for example, *P* or *vP*, and it is summarized in Table 4.

#### 2.3.1. Persistence

To estimate the persistence (*P*) of a substance in the environment we have followed the approaches summarized in Table 4 based on half-lives in water and sediment [11,23] or on the OECD *P_ov_* (overall persistence) and LRTP Screening Tool [14,22].

- *P* screening

For the *P* assessment, BIOWIN or BIOHCWIN, from the EPI Suite™ v4.0 tool [24], were used [10]. BIOHCWIN estimates the half-life prediction of petroleum hydrocarbons, whereas BIOWIN estimates the rapid aerobic biodegradation of an organic substance in the presence of mixed populations of environmental microorganisms. Following [11] the screening assignment for *P* substances occurs when:

- BIOWIN 3 < 2.2 (ultimate biodegradation timeframe is equal or greater than months) and BIOWIN 6 < 0.5 (low probability of fast biodegradation).

The SMILES (Parent SMILES) codes were used as an input for the EPI Suite modules.

- *vP* screening

For screening *vP*, the OECD *P_ov_* and LRTP Screening Tool [14,22] was employed. This tool requires the molecular weight, the octanol-water partition coefficient, *K_ow_*, the air-water partition coefficient (Henry’s law constant), *K_aw_*, and the degradation half-lives, *t_1/2_*, for soil, marine water and air. The OECD Screening Tool provides the *P_ov_* value which is the overall residence time of the chemical in the entire model system and two metrics for the LRTP: the first is the characteristic travel distance, *CTD* (km), which indicates the distance from a point source at which the chemical’s concentration has dropped to 37% (e^−1^) of its initial concentration; the second is the transport efficiency, *TE* (%), that estimates the percentage of emitted chemical that is deposited to surface media after transport away from the region of release.

**Figure 2.**
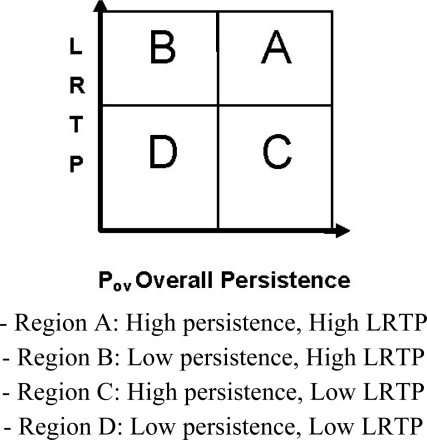
Identified region as a function of LRTP and Overall persistence [22].

The boundaries for the identification of a chemical as Persistent Organic Pollutant (POP)-like or non-POP-like are based on the values obtained for ten reference chemicals: six with high environmental half-lives and empirically known transport to remote regions, *i.e.*, PCBs 28, 101, 180; hexachlorobenzene (HCB); α-hexachlorocyclohexane (α-HCH) and carbon tetrachloride; and four chemicals with low half-lives and less pronounced (or no) occurrence at remote locations, *i.e.*, p-cresol, atrazine, biphenyl, aldrin. Using these reference values, see Table 4, four regions were identified [14] as in Figure 2:

#### 2.3.2. Bioaccumulation

Most of the approaches developed so far to estimate bioaccumulation potential, when no experimental data are available, are based on the calculation of the lipophility of the substance, sometimes using empirical correlations between a certain bioconcentration factor (*BCF*, defined as the ratio of concentrations of the chemical in the organism and in water—freely dissolved—at equilibrium) or bioaccumulation factor (*BAF*, considering also the food, *i.e.*, $\text{BAF}\;=\;\text{BCF}\;\cdot \;\prod_{i=1}^{n}\;{\text{BMF}}_{i}$, where *BMF* is the biomagnification factor expressed as the ratio of the concentration in the predator to the concentration in the diet—prey-, and *i* takes into account the trophic position in the food chain) and the octanol-water partition coefficient (*K_ow_*) for a certain organism.

According to [11] and [23], bioaccumulation assessment should be based preferably on the measurement of the bioconcentration factor (*BCF)* in aquatic species (normally fish) and the biomagnification factors *(BMF*). The criteria are:

- *BCF* > 2,000 L kg^−1^ and *BCF*< 5000 L kg^−1^ → *B*
- *BCF* > 5,000 L kg^−1^ → *vB*

In addition, if the measured *BMF* is higher than one this implies convincing evidence of bioaccumulation through the food chain [11]. The standard test to study the *BCF* in fish is the OECD 305 bioconcentration test guideline [25].

If no data are available, the substance can be considered as not *B* and not *vB* if it has a log *K_ow_* ≤ 4.5 and no specific mechanisms of uptake [11]. In addition to log *K_ow_*, non-testing data such as the molecular size (average maximum diameter and maximum molecular length), molecular weight and octanol solubility may be used in a weight of evidence approach for the assessment. Furthermore, QSARs may be used, provided that the model is appropriate for the chemical class [11].

#### 2.3.3. Toxicity

According to the REACH regulation [3], a substance is considered to fulfil the toxicity criterion (*T*) when:

- the long-term no-observed effect concentration (*NOEC*) for marine or freshwater organisms is less than 0.01 mg L^−1^, or
- the substance is classified as carcinogenic (category 1 or 2), mutagenic (category 1 or 2), or toxic for reproduction (category 1, 2 or 3), or
- there is evidence of chronic toxicity, as identified by the classifications: T, R48, or Xn, R48.

For the determination of a definitive criterion for *T*, chronic tests must be performed. The toxicity criterion (*T*) cannot be decided based on acute studies alone. A substance is considered to potentially meet the criterion for *T* classification when an acute *EC_50_* or *EL_50_* value from a standard *EC_50_* or *EL_50_* toxicity test is less than 0.1 mg L^−1^ [11]. If this screening criterion is met, the substance is referred to definitive *T* testing, and then chronic studies are required regardless of the tonnage band unless the *EC_50_* or *EL_50_* < 0.01 mg L^−1^. The standardised chronic tests on fish, *daphnia* and algae are preferred to assess the *NOEC*. In cases where no acute or chronic toxicity data are available, the assessment of the T criterion, at a screening level, can be performed using data obtained from QSARs for acute aquatic toxicity [11]. Concerning the prediction of chronic aquatic toxicity, only a few QSAR models are available and further research is necessary to increase their predictive capacities. In our case, most of them were not considered applicable for the definitive assessment of the *T* criteria.

### 2.4. Exposure Assessment

To complement the monitoring-based approach, which depends on the availability of monitoring data, with the consequent risk of false negatives by missing substances that are not subject to monitoring programmes by Member States, we have developed a complementary approach based on the use assessment, see Equation (2) and Table 2.

Since the data from ECHA/SIEFs registration process are not available yet, we have developed an algorithm to extract data from IUCLID, whereas data from SPIN database was provided by their curators [12].

The IUCLID database (latest update October 2008) contains data which were collected through an obligation put on producers and importers of high production volume chemicals and low production volume chemicals by the Existing Substances Regulation [26]. A workflow was generated to merge IUCLID with the SLoC, to calculate the sum of the production volumes for the last reported year and to extract the use and type of use of the substances.

### 2.5. Predicted Environmental Concentration (PEC) Derivation

#### 2.5.1. Multimedia model

To estimate the *PEC* values in the water compartment, the multimedia model incorporated in the OECD *P_ov_* and LRTP Screening Tool [14], has been used. Multimedia models [27] predict the distribution of a chemical between several environmental compartments. In this case, the model considers air, water and soil compartments. The model provides the percentage of distribution between these compartments on the fraction of the emitted tonnage. Then the *PEC* value in the water compartment is obtained by multiplying the annual tonnage of each substance by two parameters, see Equation (3), *i.e.*, the percentage of distribution in water in relation to soil and air provided by the multimedia model and the *Use Index* used in the Exposure assessment (see Table 2) and by dividing the result by the water volume of 25 × 10^9^ m^3^ y^−1^ suggested as an appropriate dilution factor for wide dispersive chemicals in [21].

#### 2.5.2. ECETOC TRA Tool

ECETOC has developed an integrated tool (TRA 2010 version) for calculating the exposure and related risks to consumers, workers and the environment caused by chemicals in a tiered approach:

- Tier 0: to screen chemicals and conditions of no immediate concern out of the process and to identify chemicals and conditions where further targeting risk assessment is required.
- Tier 1: based on pre-defined and conservative use scenarios corresponding to Environmental Release Categories (ERC) [21].
- Tier 2: detailed risk assessment on previously identified uses (additional more realistic exposure input).

This approach has been implemented in an Excel tool, which is freely downloadable from the ECETOC website [13]. The tool contains the user interface and the datasheets to perform risk assessment for workers and consumers and to predict the *PEC* in water, soil and sediment compartments.

In this work, we were interested only in the algorithms that estimate the environmental concentrations, and specifically the concentrations in fresh water. In this case, the minimal amount of data necessary to run the tool are reported in Table 5.

### 2.6. Predicted No Effect Concentration (PNEC) Derivation

*PNEC_aquatic_* was calculated according to [23] as Equation (4):

(4) $${\text{PNEC}}_{\text{aquatic}}=\frac{{\text{Toxicity}}_{\text{aquatic}}}{\text{AF}}$$

where *AF* refers to an Assessment Factors that depends on data availability [23]. *Toxicity_aquatic_* was calculated with preference for *NOEC* experimental data over *EC_50_* experimental data over QSAR predictions. Several databases (see next Section) were mined to find toxicological data. When no *PNEC* was accessible, the data were combined in a developed algorithm following recommendations in [23] to estimate a value for each specific substance. In case of data gaps that requested the application of QSAR, provisional *PNEC*s were calculated using the mean of the predicted *EC_50_* from the 4 developed QSAR estimation modules and *AF* = 1,000.

## 3. Results

Excel files containing the main results obtained during the model-based exercise are included in the *Supplementary Information* Section of this paper. The following files have been made available:

- *WFD_prioritisation_summary.xls* contains the SoLC list as well as relevant information on each substance.
- *WFD_Risk_Ranking_1.xls* contains the preliminary risk assessment process carried out on the SoLC.
- *WFD_IUCLID_Industry_TYPE_Use.xls* contains the information found in IUCLID and SPIN on use type and industry involved used for the calculation of *PEC*.
- *PECvsPNEC_TRATool.xls* contains the Risk ratio calculation (*PEC*/*PNEC)* using ECETOC TRA tool (version 2010) for the 78 substances assessed as category 1 in the preliminary risk assessment procedure.

### 3.1. Data Collection

Experimental data were employed whenever possible. For this reason, several databases were screened as indicated below. When no experimental data were available several algorithms to estimate physico-chemical and toxicological properties were applied and, if no method was available, QSAR approaches were specifically developed for some parts. QSAR models have been introduced in the QSAR Model Database operated by the Joint Research Centre [20,28]; in this database, QSARs are documented in accordance with OECD validation principles [29].

In particular, we have used the experimental values in EPI Suite and OECD QSAR tool [30], which access several other databases, concerning several physico-chemical properties, and we have queried Footprint [31] (chronic/acute data *NOEC*s, *EC_50_*; various taxa for pesticides), ECETOC (chronic/acute data *NOEC*s, *EC_50_*, various taxa) [32] and DSSTOX (acute toxicity data for fish, *EC_50_*) [33] for mining toxicity experimental data. Finally, to apply the ECETOC TRA tool [13], we have collected also data concerning use assessment for 827 and for 301 substances from IUCLID and SPIN [12], respectively.

### 3.2. Hazard Assessment

#### Persistence

From 2,034 substances the *P* assessment was possible for 1,869. BIOHCWIN was used for 142 substances. A *P* score of 1 was assigned to 741 substances, 41 from BIOHCWIN and 691 from BIOWIN. Moreover, the BIOWIN modules 3 and 6 were used to assess the biodegradability of substances.

For the application of the OECD P_o_*_v_* and LRTP Screening Tool [14,22] the estimation of *K_ow_* and *K_aw_* was performed using EPI Suite™ v4.0 when no experimental data was available. The water half-life was assigned based on BIOWIN 3 output using the corrections proposed by [34]. The sediment half-life was estimated doubling half-life values in water whereas air half-life was obtained using the estimated atmospheric oxidation half-life value from EPI Suite.

A preliminary screening showed that, after eliminating metals and organometallic substances, there were 16 chemicals for which the calculation was not possible because a log *K_aw_* or an air half-life was not estimated by EPI Suite. This happened because the substance was outside the validity domain of the method. In addition there were more than 130 chemicals for which the calculated values were outside the normal range considered by the software. The values for the 16 chemicals were corrected by assigning the lowest log *K_aw_* and air half-life time values found in the set. The results show that if we classify chemicals as very persistent (*vP*) those in the Region A (See Section 2.3.1), non-persistent those in Region D and intermediate those in Regions B and C, we obtain the following results:

1. vP: 138 substances (13.2%)
2. Intermediate: 346 substances (33.1%)
3. NonP: 561 substances (53.68%)

**Figure 3.**
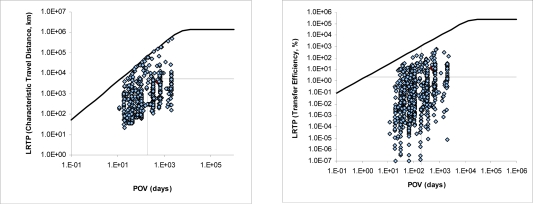
Example of results on the classification of Persistence and Long Range Transport [14] for the SLoC according to CTD (left) and TE (right). Persistent -Class A top right, Non-persistent-Class D bottom-left.

Figure 3 shows the distribution of all analyzed substances in the four classes defined in the screening tool. Similar calculations has been carried out for the list of Plant Protection Products [5] (PPP, 889 substances) and corresponding registered chemicals (6,0384 substances) with the following percentages: persistent: 6.5 and 9.1%, intermediate: 31.1 and 22.7% and non-persistent: 62.4 and 68.2%, respectively. In general terms, the results seem to agree with our expectations in the sense than the SLoC contains higher percentages of persistent chemicals indicating therefore that the preliminary selection has been done properly.

#### Bioaccumulation

After a discussion by the WG-E Working Group on Prioritisation, it was proposed to use experimental *BCF* values when available (EPI Suite contained 307 experimental data points, whereas Footprint database contained 312) and to apply QSAR models when no experimental data existed using the worst case QSAR estimated values for this screening phase.

Three modelling approaches were applied to estimate *BCF*: EPI Suite (BCFBAF), CAESAR bioaccumulation [35] and a JRC BCF model [20]. These QSAR models represent the state-of-the-art for QSAR bio-concentration models with error predictions in the range of experimental variability (0.5 log units).

In all cases, the Canonical Smiles Parent of the substances was used to generate the predictions. A *BCF_max_* was generated and used to assign a score, *BCF_mean_* and *BCF_StdDev_* were used to assess the coherence of the prediction.

#### Toxicity

As indicated previously precedence was given to chronic over acute data and to experimental data over QSAR estimation. The QSAR models were generated by the ADMET modeller software [36]. Three acute aquatic toxicity models using the 577 experimental data from DSSTOX dataset [33] (EPA fathead minnow acute toxicity database), were generated by different modeling methods (multi-linear regression, kernel partial least squares regression, artificial neural network). Additionally the ADMET predictor proprietary model for aquatic toxicity was used to assign screening scores in a consensus approach, *i.e.*, the screening assignment was *T* if 3 or 4 QSAR models classifications agree on the *T* classification.

### 3.3. Exposure Assessment

The analysis of IUCLID database using CAS numbers from SLoC produced more than 15,000 dossiers, related to 931 substances. The data collection covers data from 1990 to 2005. The use patterns were applied to generate the *use index* (see Table 2). In case of reported uses as pesticides, cosmetics and pharmaceuticals the *use index* was set to 1. The maximum and minimum use indices were calculated, but the use assessment score was based on the maximum *use index*.

Moreover, the SPIN database was also analyzed. SPIN collects data from the use of substances in products in the Nordic countries. Production volumes from 2006 and 2007 were collected, divided by 2 and multiplied by 20 (population factor) to estimate the use of substances at the European scale. Information on the industrial use of the use categories was translated to IUCLID types of uses and IUCLID uses to assign a *use index* to the substance. When no information was available from IUCLID, tonnages from SPIN were extrapolated to European scale to be comparable with IUCLID data. It is evident that this approach should be evaluated on a case by case basis. However, since we were performing a first screening and also because IUCLID data are relatively old (1999–2005), making the intercomparison between both databases difficult, it was felt that when recent data from ECHA will become accessible, after December 2010, a more accurate calculation could be performed.

The cases in which both information on monitoring and tonnage/uses were available, allowed the development of a combined single score.

### 3.4. Resulting List of Candidate Substances

A summary of the 2,034 SLoC substances as well as their physico-chemical properties is provided in the excel file: WFD_prioritization_summary.xls. The first page contains the parameters, units and definitions of the columns in the Excel file.

From the initial 2,034 substances, the risk ranging process could be performed for 737 substances. The main bottleneck in this process was the production and use data, which were not available for a considerable proportion of the substances in the SLoC. It is foreseen that with REACH more data will become available after December 2010 and therefore, the approach will cover a major number of substances. We should also highlight that IUCLID data sometimes referred to the beginning of 00’s and therefore certain values could not be representative of the actual situation.

### 3.5. Resulting List of Risk Ranked (PEC/PNEC) Substances

The main results of the model-based prioritisation are summarized in the Excel file: *WFD_Risk_ranking_1.xls*. In this file the final list ranked according the risk ratio, *PEC*/*PNEC*, for the 78 compounds classified as 1, is provided. The use of the chemical their application and the type of industrial use is provided in: *WFD_IUCLID_Industry_TYPE_Use.xls*. The related RCR (Risk Characterisation Ratio) of each substance was calculated by dividing the *PEC* by the *PNEC* value. The calculations as well as the results are reported in the Excel file: *ECETOC_application_Score1_PECvs PNEC_February2010.xls*. The results of the application of the different methodologies and tools are discussed in Section 4. Table 6 summarizes the chemicals with a Risk ratio greater than one.

## 4. Discussion

### 4.1. Assessing the SLoC Representativity

When assessing the likelihood of the list of substances to be representative of the compounds present in EU waters several considerations were made. Specifically:

- Banned Plant Protection Products (PPP): it was argued that PPP which are already banned and they are not any longer produced or placed on the European market should not be considered since risk management measures have been already taken. However, it was pointed out that looking from an ecosystem health perspective they still pose a risk, therefore, it was agreed to keep these substances in the SLoC. In addition, local authorities are monitoring, amongst others, banned pesticides to understand the effectiveness of the implementation of risk reduction measures.
- Emerging chemicals: it was emphasized that emerging substances (ES) for which less monitoring data is available should be included. The NORMAN network [16] provided the list of emerging substances.
- Pharmaceuticals: Even though European legislation managing pharmaceuticals already exists, some of these substances were included in the SLoC.
- Grouping of Chemicals: a strategy is needed for grouping chemicals for specific substances having congeners (e.g., PAH—Polycyclic Aromatic Hydrocarbons, PBDE—Polybrominated Diphenyl Ethers, PCB—Polychlorinated Biphenyls, PCDD/F—Polychlorinated Dibenzo-*p*-Dioxins and Polychlorinated Dibenzofurans). However, to take into account the combined effects of chemical mixtures would need a different approach and it was decided to run first the prioritisation process and then to study the possibility of grouping on a case-by-case basis depending on the selected compounds.

### 4.2. Toxicity Assessment

In a recent study on the application of non-testing methods to characterize chemicals [37], it was concluded that the sole reliance on QSARs to estimate acute and chronic toxicity is not recommended and toxicological data are still necessary. However, in the absence of these data a combined approach using several methodologies could be useful in a first screening phase to assess if a substance is potentially toxic.

In this work, when toxicological information was not available, several QSARs were developed to estimate toxicity for the screening of the substances as well as for the calculation of PNEC values. Figure 3 shows for example the statistical data for the QSAR model (observed/predicted *LC_50_*), generated by an artificial neural network.

### 4.3. PEC Calculation: Comparison between ECETOC TRA and OECD P_ov_ and LRTP Screening Tool

**Figure 4.**
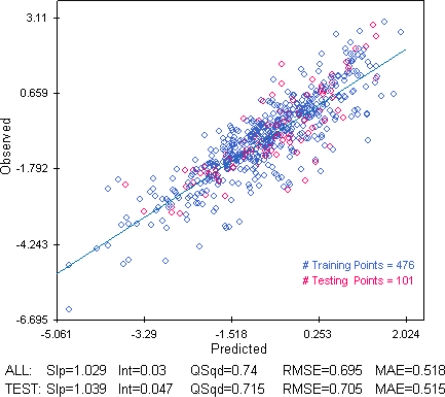
Example of an “in-house” developed QSAR to predict toxicity (LC_50_).

To compare the *PEC* results obtained using ECETOC TRA tool, we applied the values obtained from the multimedia model concerning the distribution of the compounds between air, water and soil. The hypothesis was that the values obtained using Equation (3) should be an extreme in the calculation of the *PEC*, *i.e.*, *PEC_OECD_* > *PEC_ECETOC_*. Figure 4 shows the results obtained.

As it can be observed, using the last version of the tool, the predictions are confirmed for all the ERCs (Environmental Release Categories) considered here: ERC2 (*formulation of preparations*), ERC4 (*industrial use of processing aids*), ERC5 (*industrial use resulting in inclusion into or onto a matrix*), ERC6 (a = *industrial use of intermediates*; c = *production of plastics*; d = *production of resins/rubbers*), ERC8 (a = *wide dispersive indoor use of processing aids in open systems*; c = *wide dispersive indoor use resulting in inclusion into or onto a matrix*; d = *wide dispersive outdoor use of processing aids in open systems*; f = *wide dispersive outdoor use resulting in inclusion into or onto a matrix*), ERC10 (a = *wide dispersive outdoor use of long-life articles and materials with low release*; b = *wide dispersive outdoor use of long-life articles and materials with high or intended release*).

**Figure 5.**
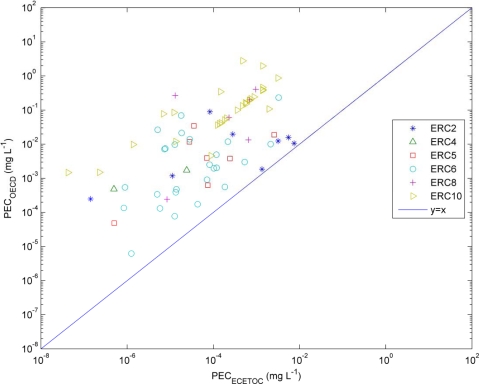
Comparison between *PEC* estimated using ECETOC and Equation (3). ERC = Environmental Release Categories (Appendix R.16.1, REACH Guidance, Chapter R.16).

For the case of ERC10, which corresponds to wide dispersive outdoor use—typical of pesticides, the relationship between the results of multimedia model and ECETOC TRA tool is practically constant by a factor of ∼280 for a high number of compounds (Figure 5), whereas for the other categories there is higher dispersion. It can be therefore concluded that the application of Equation (3) with the *use_index* defined in Table 2 may provide a worst case scenario when evaluating the environmental concentrations in water as a function of the amount of production and the intended use of the substance.

## 5. Conclusions

In this study, a modelling-based prioritisation scheme has been developed and implemented. The approach was intentionally kept separate from the monitoring-based prioritisation scheme to be able to take into account substances for which monitoring data were not available in Member States monitoring programmes, and which could pose a risk to aquatic ecosystems and to human health. However, the approach was merged with the monitoring-based prioritisation exercise in a final step by the calculation of modelled risk ratios (*PEC*/*PNEC*). In this way, results from both approaches could be compared. However, caution should be exercised since predicted environmental concentrations need to be verified experimentally beforehand.

The present approach did not consider metals and, in some cases (when experimental physico-chemical and toxicological data were not available) organometallic substances. This is due to the fact that most of the existing correlations have been developed for organic chemicals and the predictions of some properties for these chemicals are not valid using existing approaches. To consider these families of substances would have required an additional effort that was not possible with the time and resource constraints of the project, but a parallel approach could be developed. However, due to the reduced number of this type of substances, when compared with organic chemicals, a case-by-case study should be the preferred option.

Another open question concerns the treatment of mixtures. EU legislation is mainly based on single substances. However, we are always exposed to an enormous variety of chemicals through air, water, food, medicines, cosmetics, household products, *etc.*, We believe that mixture assessment is the approach to consider; however a series of issues need to be solved before a combined assessment of chemical effects is developed, between them the selection of the procedure to calculate the total toxicity of the mixture as a function of individual toxicities, e.g., concentration addition (CA) or independent action (IA), and how to exclude the possibility of synergistic effects [38]. An overview on the State of the Art of mixture toxicity has been produced recently [39], and this is probably an issue that should be tackled after the present prioritisation exercise, for substances that are part of one of the families included in the next WFD Priority Substances list.

As far as possible, the approach made use of public domain tools (e.g., EPI Suite™, OECD P_ov_ and LRTP Screening Tool, ECETOC TRA, *etc.*,) to make the approach accessible to all parties. However, this was not always possible and, in some cases, in-house models had to be developed and, in others, commercial software was used. The main reasons for this were the tight schedule of the process and the amount of information to gather and process. Automated workflows were developed using the Pipeline Pilot software since a preliminary analysis of PBT substances from the REACH PRS list had already been performed. However, open source software, e.g., KNIME [40], could also be used to develop such workflows.

A long-term objective and a future option for the next prioritisation exercises, could consist of the development of an open source tool able to re-calculate as a function of the increase of data (e.g., REACH registration, new monitoring programmes, toxicological data, etc.), or new analytical tools (e.g., multimedia models, QSAR models, *etc.*), or emergent pollutants, all the parameters to re-assess the risk ratio. This would be a coherent approach, but it would require an effort for the development of the tool and clear documentation that could be used to check and assess the validity of the results. The current exercise should be considered as a first step in this direction—a feasibility study showing that the approach is possible and worthwhile.

However, irrespective of the degree of automation in the process and the amount of information it is possible to deal with (all inventory of chemical substances could be introduced in the process when data become available), an expert review should always be the last step in all prioritisation exercises.

## Figures and Tables

**Table 1.** Exposure assessment scores, see Equation (2) for the calculation.

**Exposure score**	**Annual Use (tons)**

**0**	0–1
**1**	1–10
**2**	10–100
**3**	100–1,000
**4**	>1,000

**Table 2.** Exposure assessment scores, see Equation (2) for the calculation.

**Contribution**	**Unit/Value**	**Approach**

A. How much is produced/imported annually in EU?	Ton/year	Data from IUCLID and SPIN databases (Nordic Countries) [12]
B. What is the use pattern?	Use Index (0.1–1)	**0.1** Controlled system (isolated intermediate) **0.2** Industrial (non dispersive) use or use resulting in inclusion into/onto matrix **0.5** Wide dispersive use (mainly diffusive sources) **1.0** Used in the environment

**Table 3.** Risk scores obtained by combining the hazard and exposure assessment results.

	**Exposure assessment score**					
**Hazard Assessment**		**4**	**3**	**2**	**1**	**0**
	**4**	1	1	2	3	5
	**3**	1	2	2	3	5
	**2**	2	2	3	4	5
	**1**	3	3	4	4	5
	**0**	5	5	5	5	5

**Table 4.** *P* and *vP* assessment criteria [11,14,22,23].

**Criteria**	**Classification**

*P*	Fresh (estuarine) water *t_1/2_* > 40 day, or marine water *t_1/2_* > 60 day, or Fresh (estuarine) sediment *t_1/2_* > 120 day, or marine sediment *t_1/2_* > 180 day.
*vP*	*P_ov_* > 195 day and CTD * > 5097 km or *TE* > 2.25%.

* CTD = Characteristic Travel Distance; TE= Transport Efficiency, see Section 2.3.1 for definitions

**Table 5.** Mandatory input required by ECETOC TRA tool to estimate *PEC* in local freshwater compartment. ERC = Environmental Release Category.

ECETOC mandatory input		Measurement unit
Substance identification	IUPAC name	
	CAS number	
	Sector of Use (SU)	
Physico-chemical properties	Molecular weight	g mol^−1^
	Vapour pressure	Pa or hPa
	Water solubility	mg L^−1^
	Octanol/water partition coefficient	*K_ow_* or log*K_ow_*
	Biodegradability test result	
Environmental exposure scenario	Tonnage	tons year^−1^
	Fraction of tonnage to region	
	ERC code	

**Table 6.** Estimated *PEC*, *PNEC* for the substances with a risk ratio (*PEC*/*PNEC*) >1.

**CAS**	**Name**	***PNEC*** (mg L ^−1^)	***PEC*** (mg l^−1^)	***Risk ratio***
2921-88-2	chlorpyrifos	3.00 × 10^−6^	1.40 × 10^−3^	465
834-12-8	ametryn	3.60 × 10^−6^	8.82 × 10^−4^	245
3520-72-7	4,4′-[(3,3′-dichloro[1,1′-biphenyl]-4,4′-diyl)bis(azo)]bis[2,4-dihydro-5-methyl-2-phenyl-3H-pyrazol-3-one]	1.97 × 10^−5^	3.19 × 10^−3^	162
5567-15-7	2,2′-[(3,3′-dichloro[1,1′-biphenyl]-4,4′-diyl)bis(azo)]bis[N-(4-chloro-2,5-dimethoxyphenyl)-3-oxobutyramide]	4.93 × 10^−5^	7.48 × 10^−3^	152
5468-75-7	2,2′-[(3,3′-dichloro[1,1′-biphenyl]-4,4′-diyl)bis(azo)]bis[N-(2-methylphenyl)-3-oxobutyramide]	3.78 × 10^−5^	5.52 × 10^−3^	146
1085-98-9	dichlofluanide	1.00 × 10^−5^	1.40 × 10^−3^	140
7287-19-6	prometryn	2.00 × 10^−6^	1.83 × 10^−4^	91
886-50-0	terbutryn	2.40 × 10^−6^	1.83 × 10^−4^	76
119-47-1	6,6′-di-tert-butyl-2,2′-methylenedi-p-cresol	4.49 × 10^−5^	3.27 × 10^−3^	73
56-35-9	bis(tributyltin) oxide	9.90 × 10^−6^	6.92 × 10^−4^	70
5102-83-0	2,2′-[(3,3′-dichloro[1,1′-biphenyl]-4,4′-diyl)bis(azo)]bis[N-(2,4-dimethylphenyl)-3-oxobutyramide]	2.81 × 10^−5^	1.35 × 10^−3^	48
42576-02-3	methyl 5-(2,4-dichlorophenoxy)-2-nitrobenzoate	3.50 × 10^−6^	1.25 × 10^−4^	36
79-94-7	2,2′,6,6′-tetrabromo-4,4′-isopropylidenediphenol	8.45 × 10^−5^	2.13 × 10^−3^	25
1897-45-6	chlorothalonil	6.00 × 10^−5^	1.39 × 10^−3^	23
21725-46-2	cyanazine	8.60 × 10^−5^	1.96 × 10^−3^	23
67774-74-7	undecylbenzene	4.60 × 10^−5^	9.69. × 10^−4^	21
50-29-3	clofenotane	5.00 × 10^−6^	7.12 × 10^−5^	14
74070-46-5	2-chloro-6-nitro-3-phenoxyaniline	5.00 × 10^−5^	6.84 × 10^−4^	14
1582-09-8	trifluralin	4.00 × 10^−5^	4.84 × 10^−4^	12
2312-35-8	propargite	6.00 × 10^−5^	5.77 × 10^−4^	10
67747-09-5	N-propyl-N-[2-(2,4,6-trichlorophenoxy)ethyl]-1H-imidazole-1-carboxamide	1.00 × 10^−4^	7.45 × 10^−4^	8
115-32-2	dicofol	8.80 × 10^−5^	5.52 × 10^−4^	6
25637-99-4	hexabromocyclododecane	9.68 × 10^−5^	5.32 × 10^−4^	6
3194-55-6	1,2,5,6,9,10-hexabromocyclodecane	4.86 × 10^−4^	2.59 × 10^−3^	5
107-64-2	dimethyldioctadecylammonium chloride	1.59 × 10^−5^	8.32 × 10^−5^	5
52740-90-6	1-amino-N-(3-bromo-9,10-dihydro-9,10-dioxo-2-anthryl)-9,10-dihydro-9,10-dioxoanthracene-2-carboxamide	4.19 × 10^−5^	2.17 × 10^−4^	5
32536-52-0	diphenyl ether, octabromo derivative	4.75 × 10^−5^	2.44 × 10^−4^	5
52315-07-8	alpha-cyano-3-phenoxybenzyl 3-(2,2-dichlorovinyl)-2,2-dimethyl- cyclopropanecarboxylate	3.00 × 10^−7^	1.38 × 10^−6^	5
68442-68-2	4-(1-phenylethyl)-N-[4-(1-phenylethyl)phenyl]aniline	1.87 × 10^−5^	8.16 × 10^−5^	4
96-69-5	6,6′-di-tert-butyl-4,4′-thiodi-m-cresol	5.04 × 10^−5^	1.85 × 10^−4^	4
55283-68-6	ethalfluralin	4.00 × 10^−6^	1.22 × 10^−5^	3
1163-19-5	bis(pentabromophenyl) ether	4.29 × 10^−5^	1.18 × 10^−4^	3
52-68-6	trichlorfon	6.00 × 10^−5^	1.53 × 10^−4^	3
1912-24-9	atrazine	1.30 × 10^−3^	3.16 × 10^−3^	2
31570-04-4	tris(2,4-ditert-butylphenyl) phosphite	1.67 × 10^−5^	3.55 × 10^−5^	2
63449-39-8	paraffin waxes and hydrocarbon waxes, chloro	1.67 × 10^−4^	2.82 × 10^−4^	2
